# A five‐gene signature is a prognostic biomarker in pan‐cancer and related with immunologically associated extracellular matrix

**DOI:** 10.1002/cam4.3986

**Published:** 2021-06-14

**Authors:** Chunlai Yu, Mingliang You, Peizhen Zhang, Sheng Zhang, Yuzhu Yin, Xiao Zhang

**Affiliations:** ^1^ Bioland Laboratory (Guangzhou Regenerative Medicine and Health Guangdong Laboratory) Guangzhou China; ^2^ CAS Key Laboratory of Regenerative Biology Joint School of Life Sciences Guangzhou Institutes of Biomedicine and Health Chinese Academy of Sciences and Guangzhou Medical University Guangzhou Guangdong China; ^3^ Department of Obstetrics and Gynecology Third Affiliated Hospital Sun Yat‐Sen University Guangzhou China; ^4^ Key Laboratory of Clinical Cancer Pharmacology and Toxicology Research of Zhejiang Province Hangzhou Cancer Institute Affiliated Hangzhou Cancer Hospital Zhejiang University School of Medicine Hangzhou China

**Keywords:** ECM deposition, ECM regulator, immune cell infiltration, immunologically cold, pan‐cancer signature

## Abstract

The tumor microenvironment (TME) is related to extracellular matrix (ECM) dynamics and has a broad fundamental and mechanistic role in tumorigenesis and cancer progression. We hypothesized that ECM regulators might play an essential role in pan‐cancer attribution by causing a generic effect through its regulation of the dynamics of ECM alteration. By analyzing data from TCGA using GSEA and univariate Cox regression analysis, we found that ECM regulator genes were significantly enriched and contributed to mortality in various cancer types. Notably, UMAP analysis revealed that ECM regulator genes dominated the differences between tumor and adjacent normal tissues based on 59 or 31 pan‐survival‐related ECM gene sets. Subsequently, a five‐gene signature consisting of the predominant ECM regulators *ADAM12*, *MMP1*, *SERPINE1*, *PLOD3*, and *P4HA3* was identified. We found that this five‐gene signature was pro‐mortality in 18 types of cancer in TCGA, and validated eleven other cancer types in TCGA and seven types in the TARGET and CoMMpass databases using overall survival analysis. KEGG pathway enrichment and Pearson correlation analysis indicated that these five component genes that were correlated with specific ECM proteins involved in tumorigenesis from the ECM receptor interaction gene set. Additionally, the fitted results of a linear model were applied to strengthen the discovery, demonstrating that the five genes were correlated with immune infiltration score and especially associated with typically immunologically “cold” tumors. We thus conclude that the *ADAM12*, *MMP1*, *SERPINE1*, *PLOD3*, and *P4HA3* signature showed a close association with a pan‐cancer effect on prognosis and is related to ECM proteins in the TME which corresponding with immunologically “cold” cancer types.

## Graphical abstract

In cancer diagnosis and treatment, a pan‐cancer approach is equally important. In this study, a 5‐gene signature composed of ECM regulator genes was identified positively related to the tumourigenesis, overall survival and immune cell infiltration. This finding allowed us to identify featured mix gene markers to describe the tumour microenvironment in pan‐cancer.

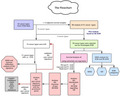

## 1 INTRODUCTION

The extracellular matrix (ECM) is a complex of fibrous, crosslinked proteins that are biochemically and biophysically crucial in the regulation of cell proliferation, survival, differentiation, and migration.^1^ In analyzing ECM structure and function, a complete “parts list” of all the proteins existing in any given matrix or contributing to matrices under different conditions are termed the “matrisome”.^2^ In mammals, the main components of the matrisome consist of almost 43 collagen subunits, 35 proteoglycans, and approximately 200 glycoproteins. In addition, there are about 176 ECM‐affiliated proteins, 250 ECM regulators, and 352 secreted factors defined as matrisome‐associated proteins associated within the core matrisome.^1^, ^3^, ^4^

Extensive research has shown that increased ECM deposition and crosslinking indicates the development and progression of cancer.^5^, ^6^ The process for controlling ECM dynamics, called ECM remodeling, is precisely regulated during development and is primarily accomplished by the expression of ECM enzymes and activities at multiple levels.^5^, ^7^ Of note, the main contributors to the activities of ECM remodeling enzymes are ECM regulators. Dysregulation or mutations in ECM regulators can result in tissue damage and even death.^8^, ^9^ For example, ECM overproduction or reduced ECM turnover resulting from ECM regulator dysfunction are prominent in fibrosis,^10^ which is related to the increased deposition of collagen I, II, III, V, and IX during tumor formation.^11^, ^12^, ^13^ It is noteworthy that ECM dynamic alterations can cause insufficient numbers of effector immune cells that have impaired ability to recognize tumor antigens or limited avidity regarding the infiltration of the tumor stroma.^14^ Recently, it was reported that ECM regulators related to ECM remodeling formed an unfavorable microenvironment to prevent immune cell penetration or serve as a barrier to anti‐cancer agents,^5^, ^15^, ^16^ which is called immunologic destruction of advanced cold tumors.

As described above, the regulation of ECM alteration in the tumor microenvironment (TME) shares a common molecular mechanism in tumorigenesis and cancer progression, which can be regarded as pan‐cancer attribution. In this respect, sustained growth and invasion of tumor tissue can be illustrated as a highly unstable escape from the primary tumor facilitated by ECM remodeling and regulated by ECM regulators, which lead to tumor tissue acquiring cellular aberrations and allowing them to travel and colonize distant organs.^17^, ^18^ Thus, we hypothesized that ECM regulators might play an essential role in pan‐cancer attribution through a generic effect on the dynamics of ECM alteration.

In this study, we conducted a comprehensive analysis of all known matrisome genes, and identified that ECM regulators served as the primary contributor correlating with overall survival and accounted for much of the difference in matrisome expression between tumor and adjacent normal tissues. Our research aims to provide a combined gene signature by using an in‐house developed pipeline, and to utilize the full spectrum of ECM‐related genes for studying the prognostic biomarker in pan‐cancer and its related genes.

## 2 MATERIALS AND METHODS

### 2.1 Data acquisition and difference analysis

The transcriptome profiles with HTSeq‐count format, mutation, methylation, and corresponding clinical information for 33 cancer types collected in The Cancer Genome Atlas (TCGA) database were downloaded from the UCSC Xena browser (https://xenabrowser.net/). Low expressing genes were filtered with a threshold mean value of log2 (count +1) <1. Differentially expressed genes (DEGs) with |log2 (foldchange)| >1 and *p* < 0.05 between the tumor and the normal adjacent tissues in 21 types of cancer with no fewer than three adjacent normal tissues were identified using the DESeq2 R package.^19^ The expression levels of signature genes were compared between primary tumors and adjacent normal tissues using Student's *t*‐test. The human matrisome gene list was obtained from the Matrisome Project (http://matrisomeproject.mit.edu). Other validated data were downloaded from the Tumor Alterations Relevant for Genomics‐driven Therapy (TARGET) and Clinical Outcomes in Multiple Myeloma to Personal Assessment of Genetic Profile (CoMMpass) studies using the UCSC Xena browser (https://xenabrowser.net/); the Chinese Glioma Genome Atlas (CGGA, http://www.cgga.org.cn/index.jsp), and three cohorts (GSE17536, GSE71187, GSE78229) from the GENE EXPRESSION OMNIBUS (GEO, https://www.ncbi.nlm.nih.gov/geo/) database. Immunohistochemistry (IHC) information and images were downloaded from the Human Protein Atlas (HPA, https://www.proteinatlas.org). Proteomic data for BRAC, READ, and OV cancer were downloaded from the Clinical Proteomic Tumor Analysis Consortium (CPTAC, https://proteomics.cancer.gov). Information on all the datasets used are listed in Table S1.

### 2.2 Gene set enrichment analysis

Gene set enrichment analysis (GSEA) based on the Kyoto Encyclopedia of Genes and Genomes (KEGG) pathways and Gene Ontology (GO) was carried out using the *ClusterProfiler* R package.^20^ GSEA based on ECM categories was performed using the *fgsea* R package.^21^ The results were visualized using the *ggplot2* R package. Statistical significance was considered when *p* < 0.05 or adjusted *p* < 0.05.

### 2.3 Survival analysis

All differentially expressed ECM (DE‐ECM) genes underwent univariate Cox regression analysis to screen survival‐related ECM (SR‐ECM) genes with a PH test: *p* > 0.05; log rank test *p* < 0.05, Wald test *p* < 0.05, hazard ratio (HR) = exp ((Expression of gene + 1) × Coefficient)/(exp(Expression of gene × Coefficient) = exp (Coefficient). Multivariate Cox regression analysis was used to construct a risk signature based on multiple genes with C_index calculated by the *survival* R package. The ROC curve plots and AUC values were obtained to validate the prognostic value of the signature using the *survivalROC* R package. The risk score for each type of cancer was calculated using the following formula: risk score = (Expression of gene_1_ × Coefficient of gene_1_) + (Expression of gene_2_ × Coefficient of gene_2_) + … + (Expression of gene_i_ × Coefficient of gene*_i_*),^22^ where *i* represents the gene index. Then, the risk score was scaled by the formula: (risk score – mean (risk score))/sd(risk score). Kaplan‐Meier survival analysis was used to evaluate the prognostic value of types of cancers; log rank test *p* < 0.05 was considered statistically significant. The HR of a signature in each type of cancer was calculated as exp(Coefficient of gene_1_ + Coefficient of gene_2_ + … + Coefficient of gene*_i_*), where *i* represents the gene index.

### 2.4 Investigation of pan survival‐related ECM genes

The pan‐survival‐related ECM (pan‐SR‐ECM) genes, which can distinguish the tumor and the adjacent normal tissues by classification of 18 types of cancer, were defined by Uniform Manifold Approximation and Projection for Dimension Reduction (UPMA) analysis combined with Principal Component Analysis (PCA) using the *umapr* R package. The value of transcripts per million (TPM) or count per million (CPM) of the selected pan‐SR‐ECM genes were used to perform PCA, and the valuable PC components were subjected to UMAP analysis.^23^ The correlation of these pan‐SR‐ECM genes was analyzed using hierarchical clustering combined with the correlation coefficients in all primary tumor tissues from the 18 types of cancer.

### 2.5 Correlation of infiltrating immune cells with the gene signature

The immune infiltration score and the abundance of 24 immune cell types from tumor tissues were evaluated using ImmuCellAI software,^24^ and single‐ and multi‐correlation coefficients with the five ECM regulator genes were calculated based on the fitted results of the linear model, multi‐correlation coefficient calculated by the formula: $\hat{y}={\hat{\beta }}_{0}+{\hat{\beta }}_{1}X_{1}+{\hat{\beta }}_{2}X_{2}+\ldots +{\hat{\beta }}_{k}X_{k}$, R=$\sum \left(y‐\bar{y}\right)\left(\hat{y}‐\bar{y}\right)$/$\sqrt{\sum ({\left(y‐\bar{y}\right)}^{2}{\left(\hat{y}‐\bar{y}\right)}^{2}}$.

### 2.6 Statistical analysis

Methylation levels of the five signature ECM regulator genes were compared between primary tumor and adjacent normal tissues by Student's *t*‐test. Mutations in these genes were compared between wild‐type and mutated‐type primary tumor tissues by Student's *t*‐test. If not specified above, a two‐sided Student's *t*‐test was performed and *p* < 0.05 was considered to be statistically significant. A flowchart for the whole analysis process is shown in Figure S1.

## 3 RESULTS

### 3.1 ECM regulator genes were significantly enriched in the matrisome across various cancer types

**FIGURE 1:**
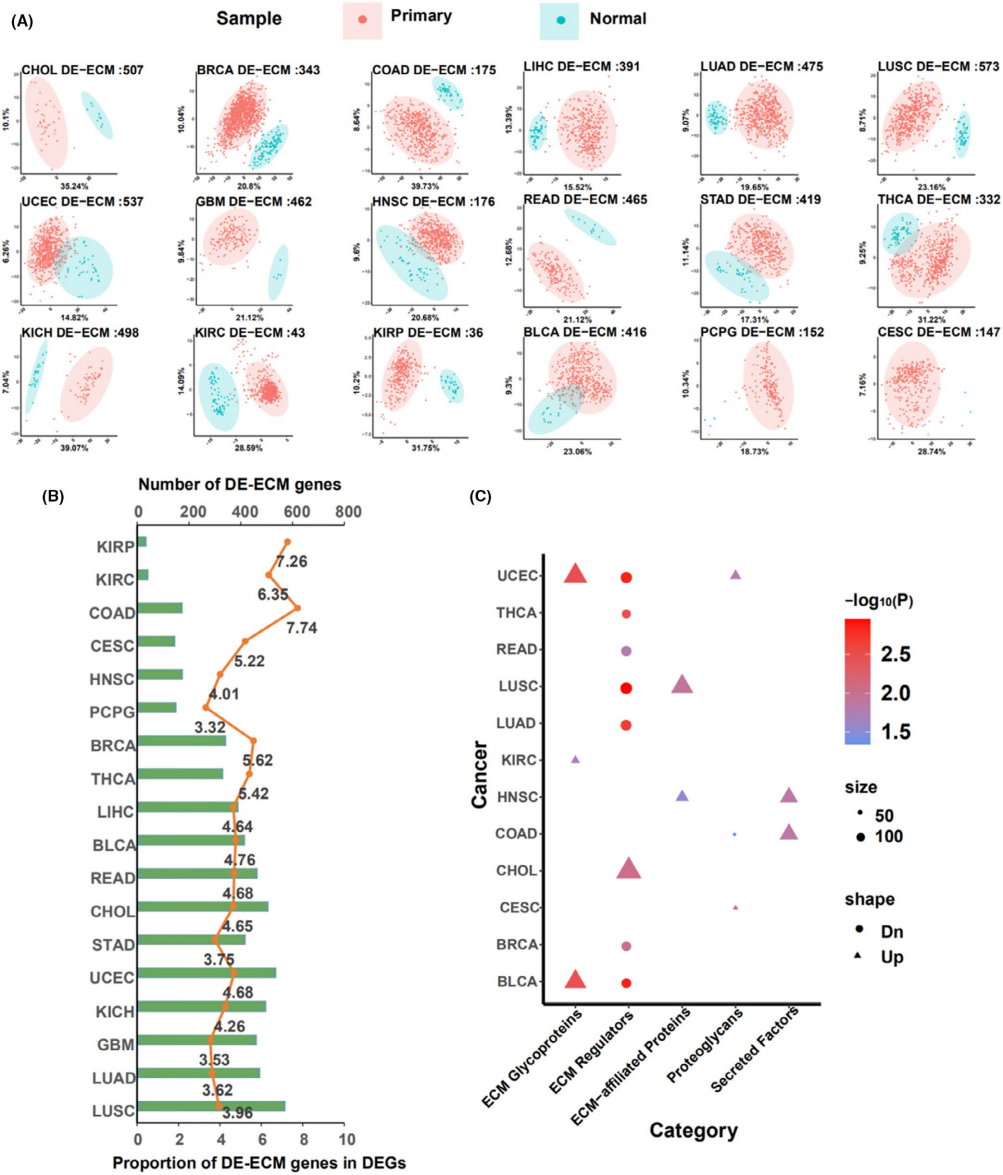
Analysis differentially expressed ECM genes across cancer types. (A) 18 types of cancer were selected by PCA based on their differentially expressed ECM (DE‐ECM) genes. Each plot presented a sample (red, primary tumor tissue or Additional ‐ New Primary; blue, adjacent normal tissue). Shadows represent the 95% confidence threshold interval. (B) Distribution of DE‐ECM genes in each cancer type. The top y‐axis shows the number of DE‐ECM genes in each type and the bottom y‐axis shows the proportion of DE‐ECM genes in DEGs. (C) Enrichment of DE‐ECM genes using the *fgsea* R package based on ECM categories. The color presents the p value; results with *p* < 0.05 are shown. Dn/Up stands for ES<0/ES >0. ES: enrichmentscore

To investigate the influence of ECM gene expression in cancer, we determined differentially expressed genes (DEGs) between tumor and adjacent normal tissues in the 21 cancer types with no fewer than three adjacent normal samples in TCGA using the *DESeq2* R package. Results obtained from PCA based on differentially expressed ECM genes (DE‐ECMs) showed that, 18 of 21 cancers types were selected as the tumor and normal adjacent tissues could be distinguished clearly. DEG analysis and the sample number of the selected cancer types demonstrated that despite only 3.53%–7.74% (mean, 4.86%) of total DEGs being DE‐ECMs, only 37 and 44 DE‐ECM genes in KIRP and KIRC, respectively, distinguished between tumor and adjacent normal tissues (Figure S2A, B). Meanwhile, KEGG and GO enrichment analysis of expressed ECM (Figure S3) revealed that the ECM genes of different cancers were involved in similar pathways, such as upregulation of cell proliferation, differentiation, and downregulation of immune cell recruitment. Subsequently, in order to identify the predominant category of ECM genes, GSEA analysis was carried out using all ECM and DE‐ECM genes based on six matrisome categories (Figure 1C, Figure S2B). The results showed that ECM regulators are significantly enriched in more cancer types (ECMs GSEA: 15 cancer types, DE‐ECMs GSEA: 8cancer types) than the other matrisome subtypes. Hence, according to GSEA analysis, ECM regulators may have a more dominant effect between tumor tissue and adjacent parts in various cancer types.

### 3.2 ECM regulator genes with a pronounced pan‐cancer mortality association

**FIGURE 2:**
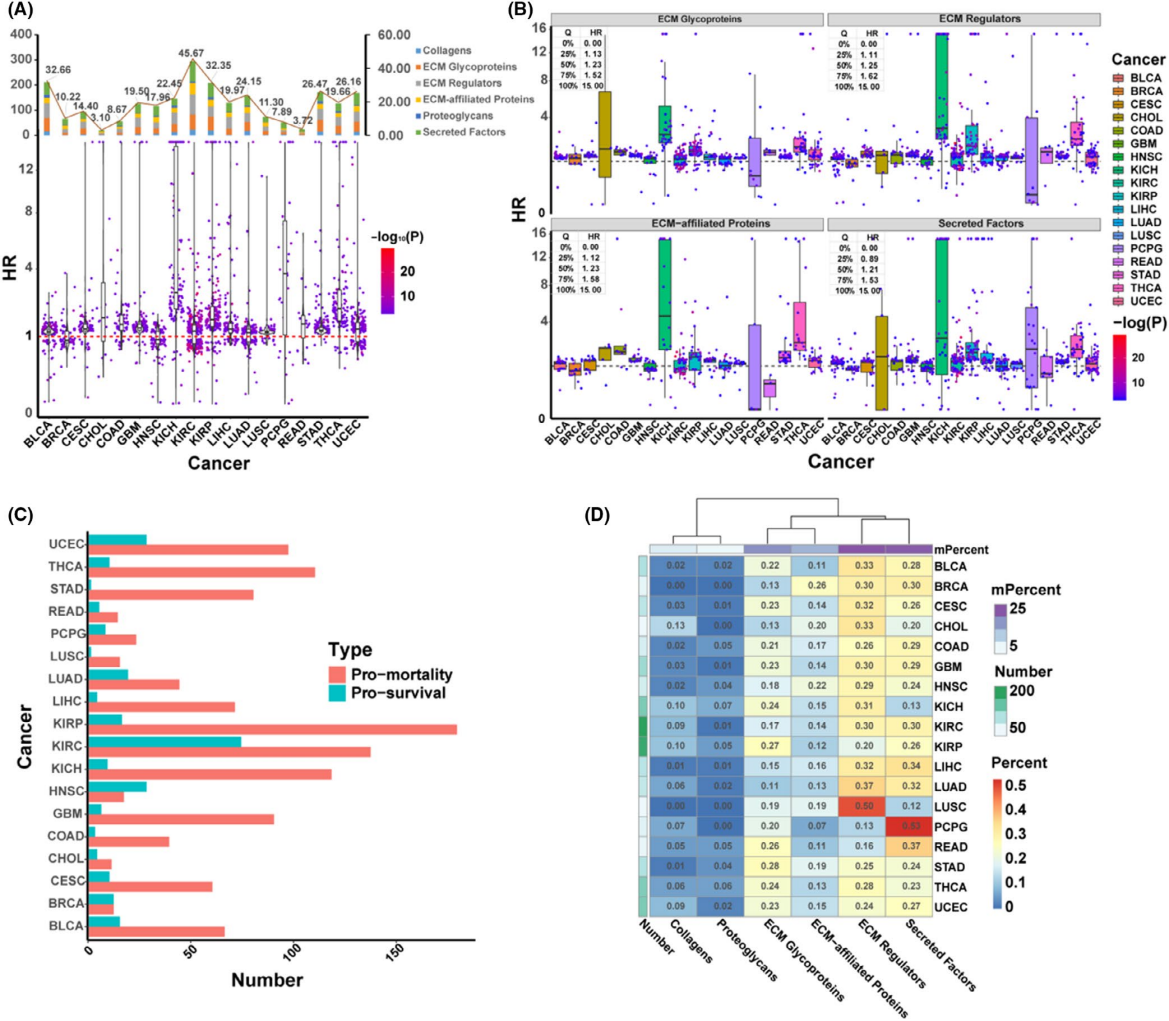
Survival‐related ECM (SR‐ECM) genes of DE‐ECM genes in 18 cancer types. (A) Distribution of SR‐ECM genes. SR‐ECM genes were screened using univariate Cox regression analysis under the control: PH test. *p* > 0.05 & Log rank test. *p* < 0.05 & Wald test. *p* < 0.05. Each plot presents a gene, genes with HR >1 are beneficial to survival, genes with HR <1 are unfavorable to survival. (B) Distribution of the four main categories of SR‐ECM genes. 16 cancer types showed a median HR >1 for ECM regulators. (C) Pro‐mortality and pro‐survival SR‐ECM genes counts in various cancers. Pro‐mortality: HR >1.2; pro‐survival: HR <0.8. (D) Distribution of pro‐mortality SR‐ECM genes categorized in ECM

Further analysis was performed to determine if a relationship existed between DE‐ECM genes and overall survival (OS). In the 18 cancer types, univariate Cox regression analysis was performed using all DE‐ECM genes, and a total of 651 survival‐related ECM (SR‐ECM) genes were found to have a statistically significant correlation. There were 16 cancer types with a median hazard ratio (HR) value >1, which implied that DE‐ECM gene expression played a significant role in the correlation with mortality (Figure 2A). The predominant groups were secreted factors (mean: 34 genes per cancer), ECM regulators (mean: 33 genes per cancer), ECM glycoproteins (mean: 28 genes per cancer), and ECM‐affiliated proteins (mean: 18 genes per cancer). In particular, ECM regulators had HR >1 in 16 of 18 cancer types and the median and upper quartile HR values were greater than other groups, which implied that expression of ECM regulators played a more critical role in mortality (Figure 2B). In addition, SR‐ECM genes were divided into pro‐mortality (HR >1.2) or pro‐survival (HR < 0.8) groups in these 18 cancer types (Figure 2C). Pro‐mortality genes appeared at significantly higher frequency than the pro‐survival group, especially in KICH, KIRP, KIRC, and THCA, which was consistent with the cancer type distribution shown in Figure 2A. Interestingly, in terms of matrisome gene categories, ECM regulators accounted for the highest proportion of pro‐mortality SR‐ECMs in 11 cancers (mean ratio: 28.56%) (Figure 2D). These findings suggest that ECM regulator genes contribute substantially to SR‐ECM genes and are correlated with a broad spectrum of cancer mortality.

### 3.3 SR‐EMC regulator genes manifest a pan‐cancer contribution in tumorigenesis

**FIGURE 3:**
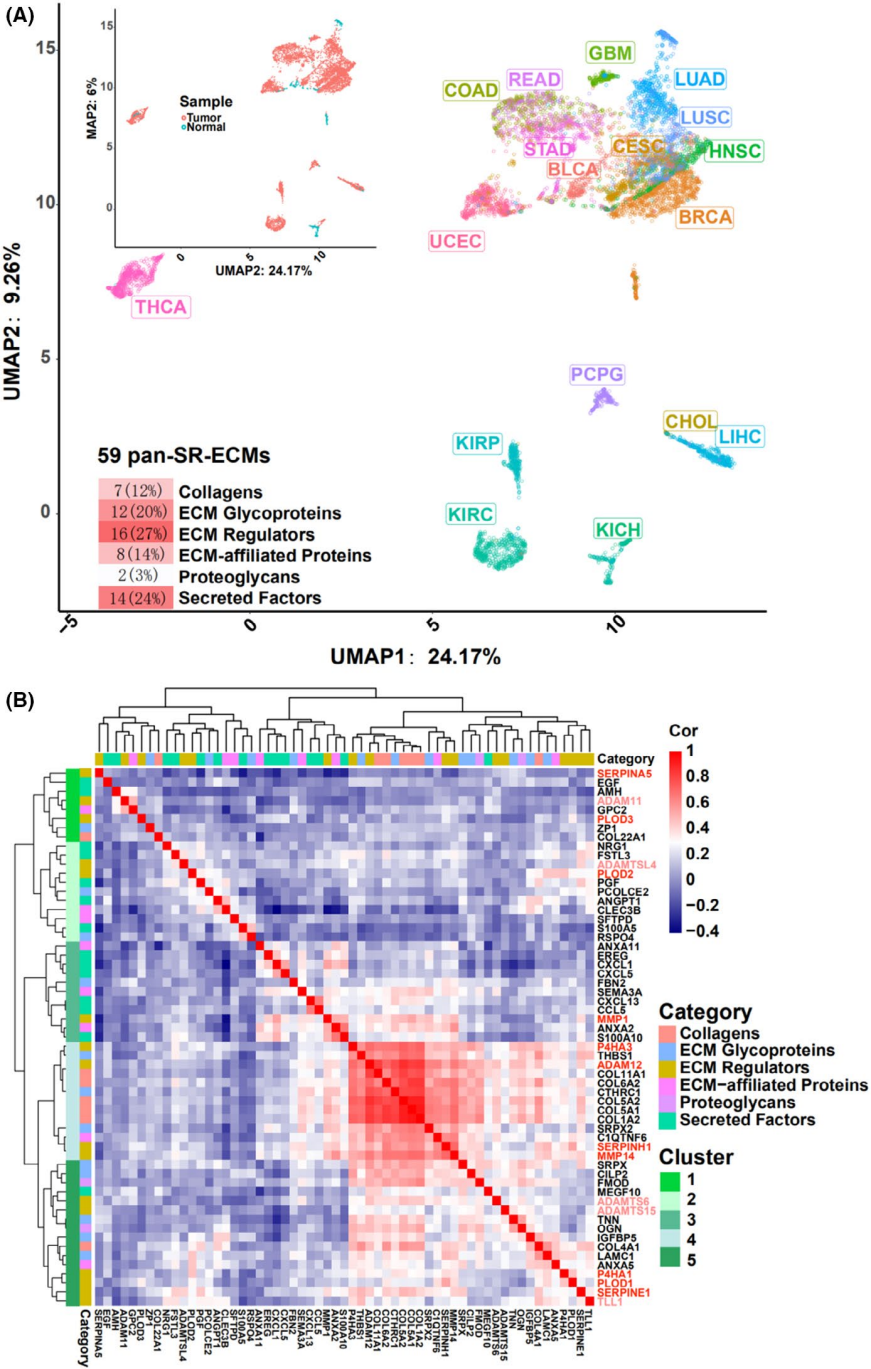
Analysis of pan survival‐related ECM (pan‐SR‐ECM) genes. (A) UMAP analysis combined with PCA based on log2(TPM + 1) value of 59 pan‐SR‐ECM genes across various cancer types. Each of the 59 pan‐SR‐ECM genes was related to survival in at least six types of cancer. (B) Correlation coefficients of 59 pan‐SR‐ECM genes analyzed using hierarchical clustering in all primary tumor tissues of 18 cancer types. TPM: transcripts per million mapped reads

As shown above, we established that ECM regulator gene expression is the primary matrisome contributor to pan‐cancer mortality. Subsequently, we investigated the correlation to pan‐SR‐ESM, especially the role of ECM regulator genes that could remodel and regulate the expression of the core matrisome. The pan‐SR‐ECM gene cluster, which could distinguish between the tumor and the adjacent normal tissues in 18 cancer types, was defined by UPMA analysis combined with PCA. We identified 59 SR‐ECM genes within at least six cancer types (Figure 3A, Figure S4), which we named pan‐SR‐ECM genes. As expected, ECM regulators accounted for the highest proportion of these pan‐SR‐ECM genes (27.12%, 16/59). These data demonstrated that organs could be specified using only these 59 pan‐SR‐ECM genes. Specifical lung (LUSC and LUAD), kidney (KICH, KIRC, and KIRP), liver (LIHC and CHOL), and intestine (COAD and READ) formed characteristic clusters. However, brain resident tumors, such as GBM and PCPG, showed a significant separation, which is consistent with an organ‐specific ECM microenvironment. As primary contributors to the difference between tumor and adjacent normal tissues, the gene expression correlation coefficients of these 59 pan‐SR‐ECM genes were further analyzed using hierarchical clustering in all primary tissues (Figure 3B). The findings showed that the 16 ECM regulator genes identified by this analysis mainly came from five gene families: a disintegrin and metalloproteinase (ADAMs)/related ADAMs with thrombospondin motifs (ADAMTS), “classical” matrix metalloproteinases (MMPs), serine proteinase inhibitors (SERPINs), procollagen‐lysine, 2‐oxoglutarate 5‐dioxygenases (PLODs), and prolyl 4‐hydroxylases (P4Hs). Genes from cluster 4 (13 genes) were strongly positively correlated with each other in all primary tissues. ECM regulator genes *P4HA3* and *ADAM12* showed a high expression correlation in cluster 4, representing a cluster of other pan‐SR‐ECM genes, including four out of six pan‐SR‐ECM collagen genes, *COL11A1*, *COL6A2*, *COL5A2*, *COL5A1*, and *COL1A2*. *MMP1* from cluster 3 was also strikingly and positively related to *ANXA2* and *S100A19*. In cluster 5, ECM regulators *P4HA1*, *PLOD1*, *SERPINE1*, and *TLL1* were clustered together and were positively correlated with each other. Collectively, using different approaches, we identified five ECM regulator families which showed great significance combined with the most pronounced pan‐cancer effect over various cancer types.

### 3.4 ADAM12, MMP1, SERPINE1, PLOD3, and P4HA3 as a five‐gene signature associated with poor PAN‐cancer survival

**FIGURE 4:**
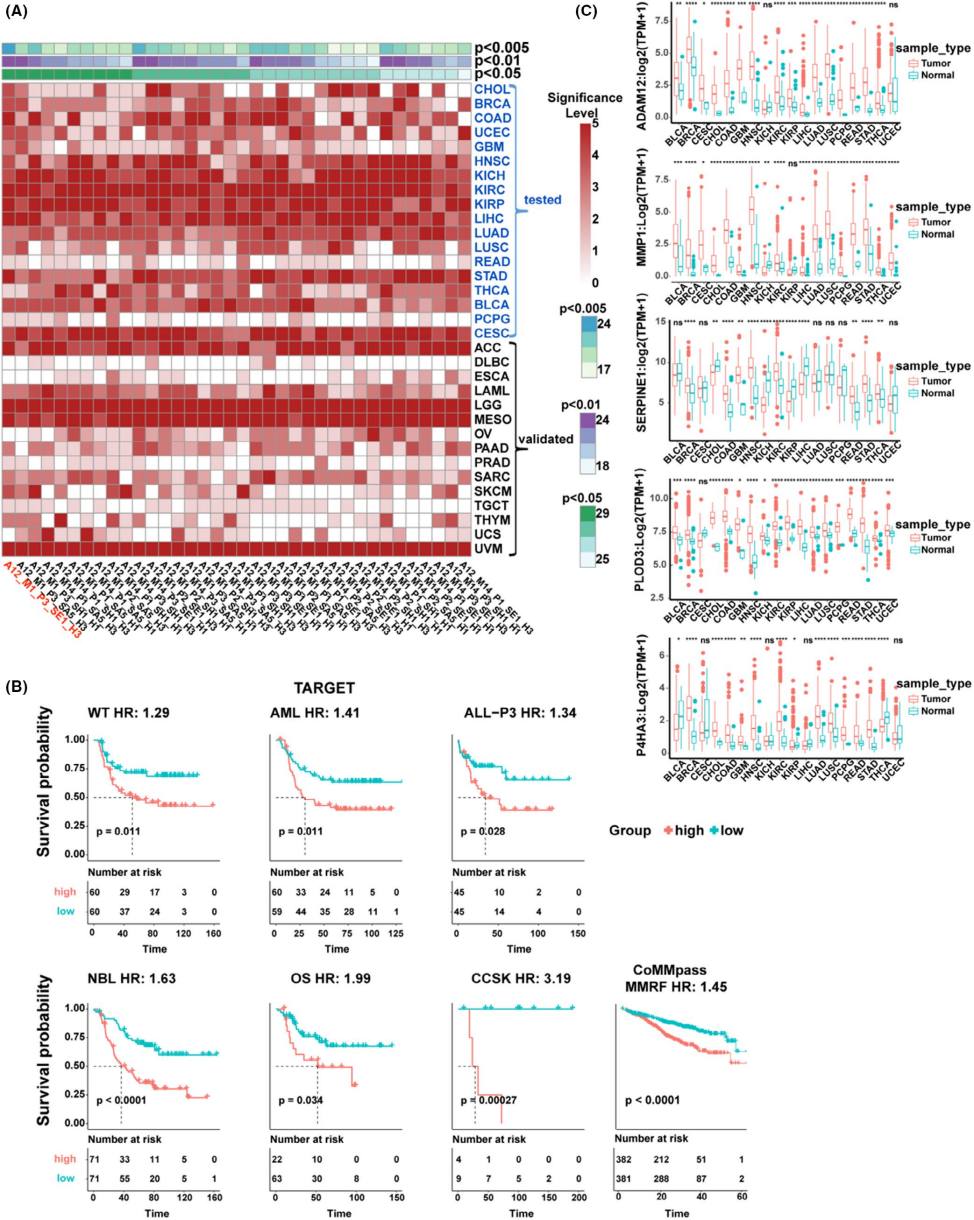
Analysis of the five regulator genes *ADAM12*, *MMP1*, *SERPINE1*, *PLOD3*, and *P4HA3* in pan‐cancer. (A) Results of different signatures consisting of 11 pan‐SR‐ECM regulators from the ADAM, MMP, SERPIN, PLOD, and P4H gene families using Kaplan‐Meier survival analysis in various cancer types from TCGA. The 18 cancer types of cancer tested with another left 15 types use for validation. A: ADAM, M: MMP, S: SERPIN, P: PLOD, H: P4HA. Statistical significance is depicted as: 0: *p* ≥ 0.05, 1: *p* < 0.05, 2: *p* < 0.01, 3: *p* < 0.005, 4: *p* < 0.001, 5: *p* < 0.0001. (B) Kaplan‐Meier plot curves of the *ADAM12*, *MMP1*, *SERPINE1*, *PLOD3* and *P4HA3* five‐gene signature validated in other cancer types from other databases. The p value was calculated using the log‐rank test. HR was calculated using the Cox proportional‐hazards model based on scaled risk score. (C) Expression levels of *ADAM12*, *MMP1*, *SERPINE1*, *PLOD3*, and *P4HA3* in the tumor and adjacent normal tissues across 18 cancer types. TPM: transcripts per million mapped reads. Statistical analyses were performed using the unpaired *t*‐test (*p < 0.05, **p < 0.01, ***p < 0.001, ****p < 0.0001)

Since the aim of this study was to identify a representative and multiple gene signature describing pan‐biological functional and pan‐survival ability, we selected one gene each from the five gene families, which consisted of 11 total pan‐SR‐ECM regulator genes that were survival‐related to at least seven types of cancer. Collectively, this produced 36 types of signatures. The coefficient of *PLOD3* made a greater contribution than the other ten ECM regulator genes using the least absolute shrinkage and selection operator (LASSO) Cox regression model in the 18 cancer types (Figure S5A). Based on the previous analysis, we speculated that a potentially promising five‐gene signature would be composed of *P4HA3* and *ADAM12* from cluster 4, *MMP1* from cluster 3, *PLOD3* from cluster 1, and *SERPINE1* from cluster 5. In the 18 cancer types tested, and another 15 validated cancer types, multivariate Cox regression analysis was performed to obtain the scaled risk score of these 36 possible signatures, following which the samples were divided into high and low groups according to the scaled risk score to conduct Kaplan‐Meier pan‐cancer survival analysis (Figure 4A, Table S2). As expected, all signatures were prominently survival‐related in pan‐cancer, and the five‐gene signature consisting of *ADAM12*, *MMP1*, *SERPINE1*, *PLOD3*, and *P4HA3* was survival‐related in the most types of cancer under different p threshold values (*p* < 0.05, 29 types of cancer; *p* < 0.01, 24 types; *p* < 0.005, 24 types; mean C_index = 0.65, mean AUC value = 0.68). Moreover, in order to carry out the evaluation process, the five‐gene signature was validated in seven different cancer categories using the independent databases TARGET and CoMMpass (Figure 4B). This result agreed that this five‐gene signature was broadly associated with poor survival in various cancer types, which was further consolidated under the number of cancer types in multiple databases for verification purposes (Figure S5B). The survival analysis results based on these cancer types were also examined using the ROC curve method (Figure S5C‐D). Moreover, survival analysis based on the signature genes was verified using CPTAC clinical proteomics datasets, which showed that the gene products were related to survival, as expected (Figure S6).

Further analysis of differences in the gene expression levels of *ADAM12*, *MMP1*, *SERPINE1*, *PLOD3*, and *P4HA3* between tumor and adjacent normal tissues in 18 cancer types from TCGA were examined (Figure 4C). The results showed that the expression levels of these five regulators were significantly different across most types of cancer. In detail, both *ADAM12* and *MMP1* expression were upregulated in 16 cancer types (*p* < 0.05), *PLOD3* was upregulated in 17 cancer types (*p* < 0.05). As a comparison, *SERPINE1* was upregulated in 8 out of 12 cancer types (*p* < 0.05) and *P4HA3* was 12 of 14 cancer types (*p* < 0.05). This finding indicates considerable upregulation in these five genes in cancers, which is consistent with the expression pattern of most ECM regulators in most of the tumor tissues. The findings were further verified using IHC staining slides from the Human Protein Atlas. As a result, the higher protein expression levels of ADAM12, PLOD3, P4HA3, and SERPINE1 were detected in multiple types of cancer compared with the normal tissue specimens, which was consistent with their mRNA expression patterns (Figure S7). The protein expression levels of PLOD3 and P4HA3 were high in most cancer tissues. MMP1 was not listed in the database, and ADAM12 showed limited detection. Interestingly, SERPINE1 demonstrated minimal detection level in most tumor tissues, but as suggested in the database, it is generally positive in the tumor‐related stroma population.

To determine whether the expression level was the primary factor associated with clinical relevance, gene mutation and methylation status were investigated. In the 18 cancer types from TCGA, we calculated tissues with mutations of *ADAM12*, *MMP1*, *SERPINE1*, *PLOD3*, and *P4HA3*, and they had limited mutations, and their mutation sites had few significant effects on gene expression or function gain across the 18 cancer types (Table S3, Figure S8A–D). In contrast, multiple methylation sites demonstrated a substantial impact on the expression of these five genes (Figure S9, Table S4), especially *PLOD3*, *MMP1*, and *ADAM12*, most of which were downregulated in pan‐cancer tumor tissues compared with adjacent normal tissues. These data demonstrate that the expression levels of these five genes have universal effects on many cancers.

### 3.5 Expression of ADAM12, MMP1, SERPINE1, PLOD3, and P4HA3 in an ECM‐receptor interaction gene set correlated with immune cell infiltration

**FIGURE 5:**
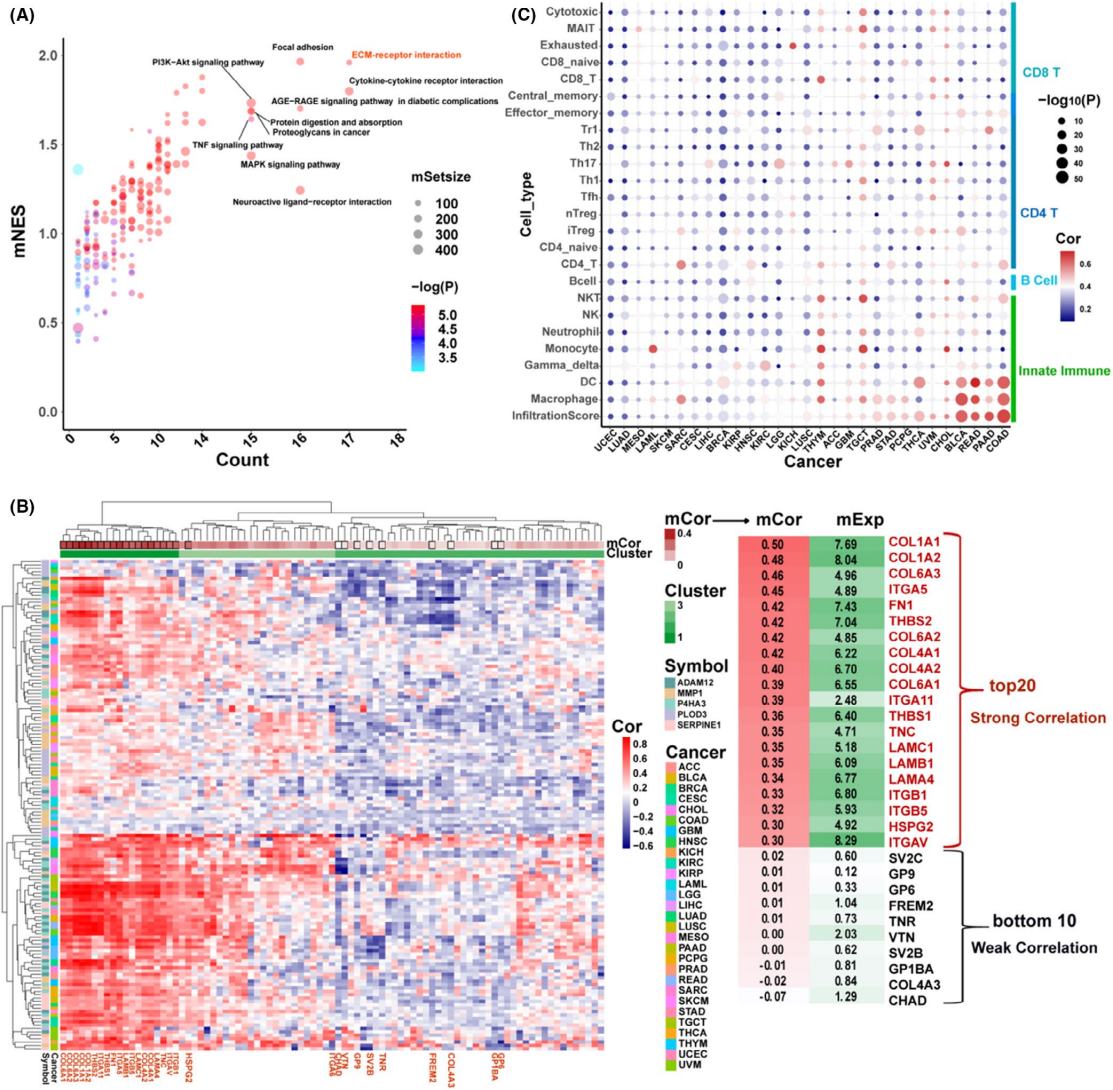
(A) Distribution of the significantly enriched KEGG pathways with mNES > 0; *x* axis: the KEGG pathway count with *p*.adjust < 0.05, *y* axis: mNES, medium value of normalized enrichment score. Each plot is a KEGG pathway, coloring based on minimum p.adjust. (B) The correlation of *ADAM12*, *MMP1*, *SERPINE1*, *PLOD3*, and *P4HA3* with the ECM‐receptor interaction gene set. (C) The multiple correlation coefficients of *ADAM12*, *MMP1*, *SERPINE1*, *PLOD3*, and *P4HA3* with infiltration score and the abundance of types of immune cells. The list was ordered by the correlation coefficient with infiltration score. Results with *p* < 0.05 are shown

To understand the mechanistic aspect of the five‐gene signature, KEGG pathway enrichment analysis between the high‐ and low‐scaled risk score groups was performed in 29 cancer types. The number of significantly enriched pathways (adjusted *p* < 0.05) with a mean value of the normalized enrichment score (mNES) was counted (Figure 5A, Figure S10A). The common proliferation‐ and apoptosis‐related PI3K‐Akt and MAPK signaling pathways were listed in 15 and 16 of the 29 cancer types, respectively. Gene sets directly related to ECM included ECM‐receptor interaction and focal adhesion were especially enriched in most cancers with the highest mNES scores. The immune response‐related gene sets cytokine‐cytokine receptor interaction and TNF signaling pathway were also in the top three clusters.

The ECM‐receptor interaction gene set was further analyzed for correlation with *ADAM12*, *MMP1*, *SERPINE1*, *PLOD3*, and *P4HA3* in the 29 cancer types. The heatmap shows that the ECM‐receptor interaction genes were grouped into three clusters, most of which had consistent expression levels (Figure 5B and Figure S10B). We found that cluster1 contained 19 genes that were highly correlated with expression levels of *ADAM12*, *MMP1*, *SERPINE1*, *PLOD3*, and *P4HA3* and had higher expression levels as a whole. The top 20 genes with the strongest or top 10 genes with the weakest correlation are presented in Figure 5B. In the top 10 most substantially correlated genes, the collagen family occupied 7 of 10 genes, and *COL1A1* was at the top of the list. In comparison, the weakest related genes showed a much more diversified ECM‐related receptor profile (Figure 5B). This finding might imply the function of *ADAM12*, *MMP1*, *SERPINE1*, *PLOD3*, and *P4HA3* and the molecular properties of the tumor tissue.

Next, we used ImmuCellAI to evaluate the immune infiltration score and the abundance of 24 immune cells from tumor tissues, and then calculated the multiple and single correlation coefficients of *ADAM12*, *MMP1*, *SERPINE1*, *PLOD3*, and *P4HA3* with mRNA expression and immune infiltration score (Figure 5C, Figure S10C–H). In general, the mRNA expression levels of *ADAM12*, *MMP1*, *SERPINE1*, *PLOD3*, and *P4HA3* were correlated with immune infiltration score and macrophages and dendritic cells (DCs) in most types of cancer. Specifically, the top three most pronounced cancer types were typical immunologically “cold,” such as COAD, PAAD, and READ. Interestingly, the data illustrated that COAD, PAAD, and READ were highly related to macrophage infiltration with less pronounced CD8+ T cell count; this finding was corroborated using TARGET and CoMMpass (Figure S5B). This observation suggests that *ADAM12*, *MMP1*, *SERPINE1*, *PLOD3*, and *P4HA3* are related to the immunological status of the ECM environment in various cancer types.

## 4 DISCUSSION

The pan‐cancer prognostic signature usually involves five clusters: cell cycle phase, immune response, cell adhesion, metabolic process, and gene expression regulation.^25^ As reported elsewhere, tumorigenesis and progression are correlated with increased ECM deposition and crosslinking, consistent with all pan‐cancer effects listed. Several studies have provided evidence that the ECM contributes to cancer pathogenesis, where ECM regulators are involved in (a) stimulating intracellular signaling that promotes invasion and proliferation; (b) promoting and creating a favorable microenvironmental niche for metastasis; (c) interfering with the communication between cancer and immune cells; and (d) forming an unfavorable microenvironment to prevent immune cell penetration or act as a barrier to anti‐cancer agents.^5^, ^15^, ^16^ These results imply that ECM dynamic regulation is related to a much broader spectrum of ECM composition. Here, we discovered that ECM regulator genes were significantly enriched in various cancer types, which also contributed to mortality and differences in 18 different cancer types. The most predominant ECM regulators, *ADAM12*, *MMP1*, *SERPINE1*, *PLOD3*, and *P4HA3*, have been classified as a five‐gene signature that produces a broad effect over 29 types of cancer. This effect is related to ECM receptor interaction and other tumor proliferation gene sets, indicating its association with impaired immune infiltration score. This regulation of ECM dynamics is also consistent with other pan‐cancer hallmarks, such as telomerase‐associated and epithelial‐to‐mesenchymal transition (EMT) signatures, which implicate a fundamental effect of regulating cell fate changes in tumorigenesis and tumor progression, such as cell‐cell contact, cellular polarity, metabolic processes, and gene expression regulation.^26^, ^27^, ^28^, ^29^, ^30^ Our results demonstrated that this five‐gene signature is highly representative of alterations to core ECM genes, which is broadly linked to tumorigenesis, and hence can be a definitive collection of prognostic biomarkers.

This five‐gene signature can represent a broad spectrum of ECM proteins in tumorigenesis and be used in a diagnostic manner. The ECM provides the functional composition to construct the tumor microenvironment (TME) and facilitates and plays critical roles during multiple stages of tumorigenesis. Increased deposition and crosslinking of ECM proteins contributes to fundamental biochemical and biophysical conditions to facilitate cancer cell proliferation, migration, and invasion.^31^, ^32^, ^33^, ^34^ Therefore, as the primary contributor to ECM dynamics, ECM regulators are involved in controlling ECM protein deposition and crosslinking. This essential TME effect has a mechanistic advantage in discovering pan‐cancer prognostic biomarker markers. Indeed, we found in this study that UMAP analysis combined with PCA showed that these 59 pan‐selected genes (≥ 6 cancer types) enabled us to distinguish tumor tissues from adjacent normal tissues at tissue‐specific resolution (Figure 3A, Figure S4C). This is consistent with the more stringent criteria that only 31 pan‐selected genes (≥7 cancer types) genes with 11 ECM regulator genes could differentiate tumor and adjacent normal tissues across 18 cancer types (Figure S4D).

ECM degradation plays an essential role in cancer cell proliferation, migration, invasion, and angiogenesis. *ADAM12* is a member of the disintegrin and metalloproteinase family and contains both a metalloproteinase and a disintegrin domain.^35^ It is involved in tumor progression, tumor cell escape, and long‐distance transformation in both orthotopic and transgenic models, which cause tumor cell proliferation, migration, and invasion^36^, ^37^, ^38^ and induces tumor cell resistance to apoptosis.^39^ *ADAM12* was also reported as a novel regulator in tumor angiogenesis via STAT3 signaling and as a prognostic marker for various cancers,^40^ including breast and prostate cancers.^41^ Similarly, MMPs are proteinase enzymes that proteolytically digest different protein substrates, including other proteinases, growth factors, cell‐surface receptors, and many components of the ECM. *MMP1* is upregulated in various cancers and has been reported to be associated with tumor invasion and metastasis,^42^ which is significantly negatively correlated with cancer survival.^43^ In contrast, overexpression of *MMP1* has been reported in different cancer types, making a path in the tumor‐associated microenvironment and contributing to cancer cell invasion.^44^, ^45^ These findings agreed with our observation that the expression levels of these two proteinase regulators’ function related to various collagen genes in a pan‐cancer manner (Figure 3B, Figure 5B).

Furthermore, our observations agree with the literature that ECM composition is regulated by a category of ECM modification enzymes, including P4Hs and PLODs. In addition, components of collagen deposition during wound healing were observed in hypoxia in tumor tissue.^46^, ^47^, ^48^ This regulation and modification were processed via enzymes such as P4H, PLOD, and LOX.^49^, ^50^, ^51^ Noteworthy expression of the P4H family is significantly upregulated in breast cancer, whereas downregulation of P4HA causes inhibition of mammary tumor growth and metastasis to the lungs, which agrees with decreased P4HA activity depresses cancer cell arrangement along collagen fibers.^52^, ^53^, ^54^ Similarly, PLOD family expression is also associated with a risk of mortality in breast cancer patients, which indicates metastasis to lymph nodes and lungs.^55^ This finding suggests that increasing fibril collagen formation increases tumor elasticity. On the other hand, the role of *SERPINE1* (*PAI*‐*1*) is less clear. *In vitro*, SERPINE1 prevents excessive pericellular degradation of ECM proteins necessary for cell adhesion and migration. It functions in modulated cell migration by both promoting cell detachment from core ECM proteins and by preventing excessive pericellular ECM degradation.^56^ In contrast, *in vivo* studies of *SERPINE1* in metastasis have reported conflicting results, as some experiments suggested a pro‐metastatic effect^57^, ^58^, ^59^, ^60^, ^61^, ^62^, ^63^ while others suggested either an inhibitory role^64^, ^65^, ^66^ or an absence of effect on metastasis.^67^, ^68^

Interestingly, we demonstrated that this five‐gene signature showed a significant correlation in cancer progression in a broad spectrum of cancer types (Figure 4A–B). This finding was verified in multiple databases at the gene expression level and CPTAC clinical proteomics datasets and was further confirmed in IHC slides from the Human Protein Atlas (Figure S6 and S8). Although the cross‐omics comparison cannot directly recapitulate gene expression levels, these findings suggest that the five‐gene signature can produce different kinds of combinatory arrangements. This maybe because the five‐gene signature might have a cooperative mechanism, which might not be related to co‐expression but provides an additive function in tumorigenesis.

The five‐gene signature‐related ECM proteins are associated with the TME. Enhanced expression of this mixed signature was related to poor survival and was associated with pan‐cancer mortality. This can be explained by the fact that these signature regulator genes might enhance deposition and crosslinking, which alters the biochemical and biophysical conditions of the TME, which leads to cell proliferation, migration, and invasion.^31^, ^32^, ^33^, ^34^ KEGG enrichment analysis of the five‐gene signature suggests underlying mechanisms in the relationship between gene clusters (Figure 5A). The gene sets with the broadest effect over 17 different cancer types enriched in ECM‐receptor interaction and cytokine‐cytokine receptor interaction gene sets. The second tier, in 16 types of cancer, was enriched in focal adhesion, which is consistent with previous reports that cancer‐derived ECM proteins (fibronectin, collagen, and laminin) are reported to protect cancer cells from chemotherapy‐induced apoptosis via activation of the PI3K/AKT pathway.^64^, ^65^

The ECM‐receptor interaction gene cluster has a broad effect, which is highly relevant to this five‐gene signature in the tumor microenvironment. Interestingly, the top 20 genes from this cluster contained a TME‐specific ECM receptor from the tumor stroma (Figure 5B). This is consistent with investigations of the matrisome in colon, lung, and breast cancer tissues,^69^, ^70^ suggesting that abnormal collagen deposition in tumor stroma leads to cancer progression, especially increased collagen VI depositions that stimulate cancer cell proliferation.^48^, ^71^, ^72^ A bulk of the tumor ECM is only expressed by cancer cells, namely *LAMA4* and *LAMB1*.^60^, ^69^, ^70^ Nevertheless, *THBS1*, *FN1*, *TNC*, *COL1*, and *COL4* were expressed in both stroma and cancer cells.^63^ Recently, it was reported that stromal cells in micro‐dissected human breast tumors expressed the most pronounced level of collagen crosslinking enzymes. This suggests that targeting these enzymes may have therapeutic potential.^73^ Moreover, other evidence suggests that genes in this cluster, such as the integrin gene *ITGAV*, were associated with higher progression and spread of various cancers via perineural invasion.^74^ In contrast, the ECM receptor *FRME2* demonstrated a weak correlation with the five‐gene signature. This observation implies that these regulators are unfavorable to conventional embryo development before birth as the FRAS/FREM complex before birth. This observation indicates that the five‐gene signature‐associated ECM contributes to the TME. Collectively, these findings can explain the underlying mechanism by which tumor modality correlates with the expression of the five‐gene signature.

The five‐gene signature implied immunogenicity in cancer for immune checkpoint‐related treatment. As reported elsewhere, the TME contains the cellular components of the tumor stroma, which include fibroblasts, endothelial cells, adipocytes, and immune cells. Cancer‐associated fibroblasts produce and regulate ECM remodeling in the TME, suggesting that cancer cells might play a role in ECM deposition. Moreover, researchers have utilized nanoparticles to apply MMP2 enzyme activity to reduce ECM deposition in the tumor microenvironment, which results in the enhancement of immune infiltration.^75^, ^76^, ^77^ This molecular operation confirmed that modulating tumor‐related ECM deposition and crosslinking could convert “cold” tumors into “hot” tumors via the potentiation of immune infiltration through less ridged ECM. Consequently, overcoming the deficiency of natural T cell responses to tumor cells, as well as T cell immunity resistance within the TME, are two critical challenges to any anti‐tumor T cell‐related therapy. More evidence has showed that in anti‐PD‐1/PD‐L1 monotherapy, the response rates which are typically around 25% and can be as low as 5% in immune‐deserted (or immunologically “cold”) tumors, such as uveal melanoma, where T cells are almost completely inattentive to the tumor.^78^, ^79^, ^80^ The evaluation of the five‐gene signature‐related immune infiltration score ranked 29 types of cancer according to the correlation. The most significantly correlated were READ (colon adenocarcinoma) and COAD (rectum adenocarcinoma), which are typically immunologically “cold” tumors, and KICH (kidney chromophobe) which was listed as a weak “cold” tumor.^81^ Hence, the five‐gene signature might provide a new insight for understanding T cell infiltration in terms of ECM deposition and crosslinking.

## 5 ETHICS STATEMENT

All data of this study were public and required no ethical approval.

## CONFLICT OF INTEREST

The authors declare that they have no conflicts of interest.

## AUTHOR CONTRIBUTIONS

SZ, XZ and YCL conceived the project, YCL, SZ and YML designed the research. YCL performed analysis. YCL and XZ co‐wrote the final text. SZ, YZY, PZZ and, provided technical assistance. XZ and YZY revised and approved the final manuscript.

## Supporting information

Table S1‐S4

Figure S1‐S10

## Data Availability

All data generated and analyzed during this work are included in the method section.
