# Supplementary material for: A five‐gene signature is a prognostic biomarker in pan‐cancer and related with immunologically associated extracellular matrix
Source: Cancer Med. 2021 Jun 14;10(13):4629–43. doi: 10.1002/cam4.3986 (PMC8267129; doi:10.1002/cam4.3986)
Supplement: Supplementary file 2 — Figure S1‐S10 [file CAM4-10-4629-s002.docx]

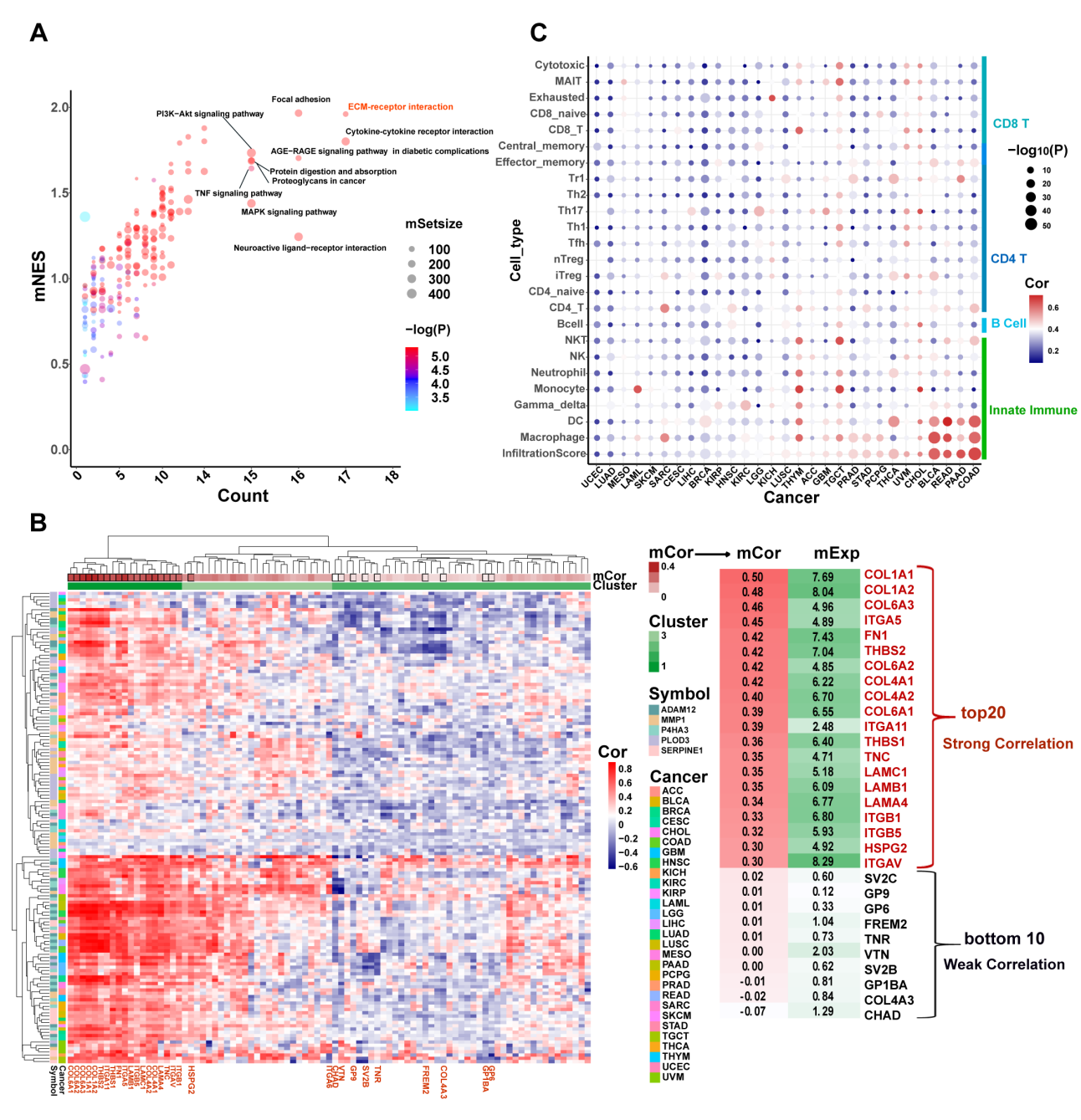


**Figure 5**. (**A)** Distribution of the significantly enriched KEGG pathways with mNES > 0; x axis: the KEGG pathway count with p.adjust < 0.05, y axis: mNES, medium value of normalized enrichment score. Each plot is a KEGG pathway, coloring based on minimum p.adjust. (**B)** The correlation of *ADAM12*, *MMP1*, *SERPINE1*, *PLOD3*, and *P4HA3* with the ECM-receptor interaction gene set. (**C)** The multiple correlation coefficients of *ADAM12*, *MMP1*, *SERPINE1*, *PLOD3*, and *P4HA3* with infiltration score and the abundance of types of immune cells. The list was ordered by the correlation coefficient with infiltration score. Results with p < 0.05 are shown.

# Supplementary data





**Supplementary Figure 1.** Analysis flowchart.


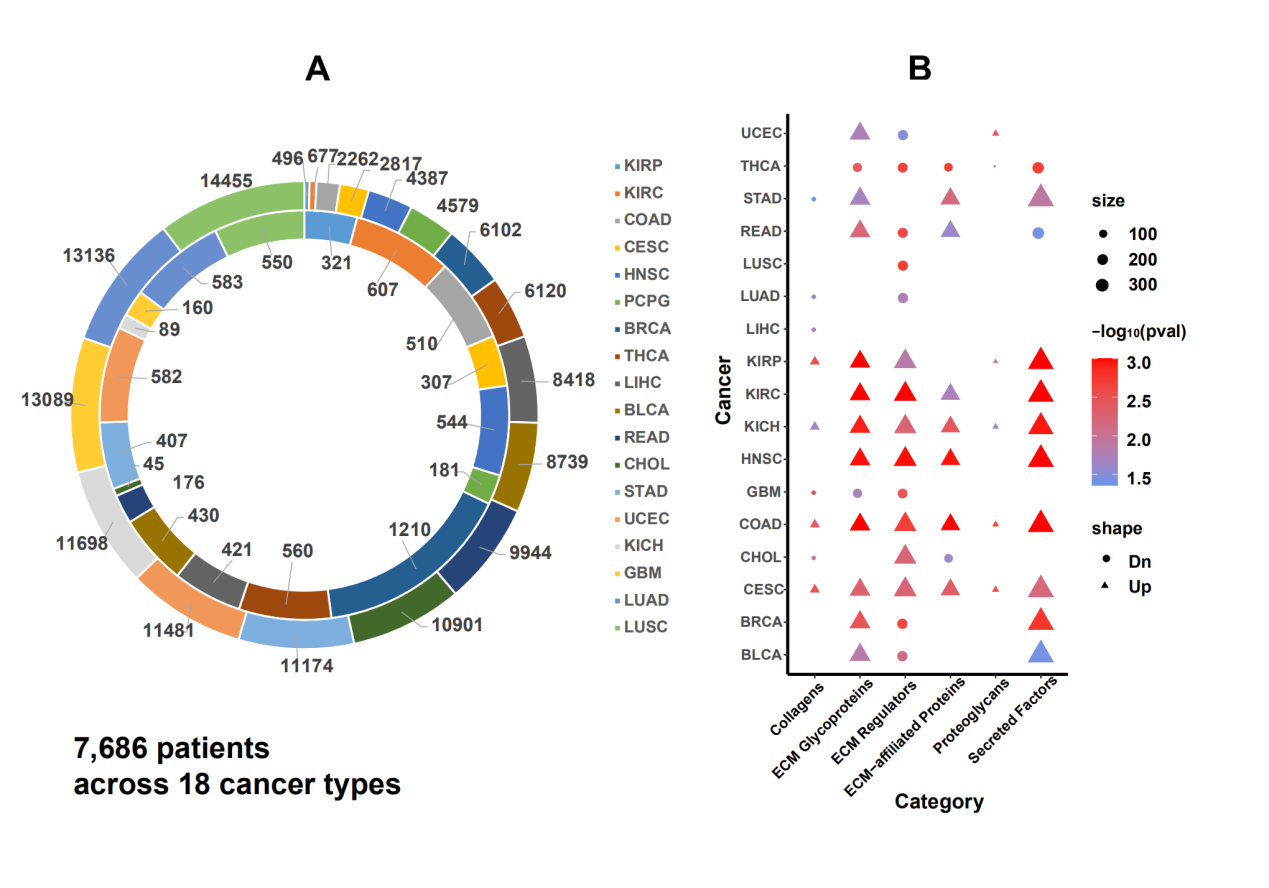


**Supplementary Figure 2.** (**A**) The number of patients and differentially expressed genes (DEGs) in each cancer type presented in inner and outer ring, respectively. (**B**) ECM genes enriched in ECM categories using *fgsea* R package. The results with p < 0.05 were showed, Dn: enrichmentscore < 0, Up: enrichmentscore > 0.


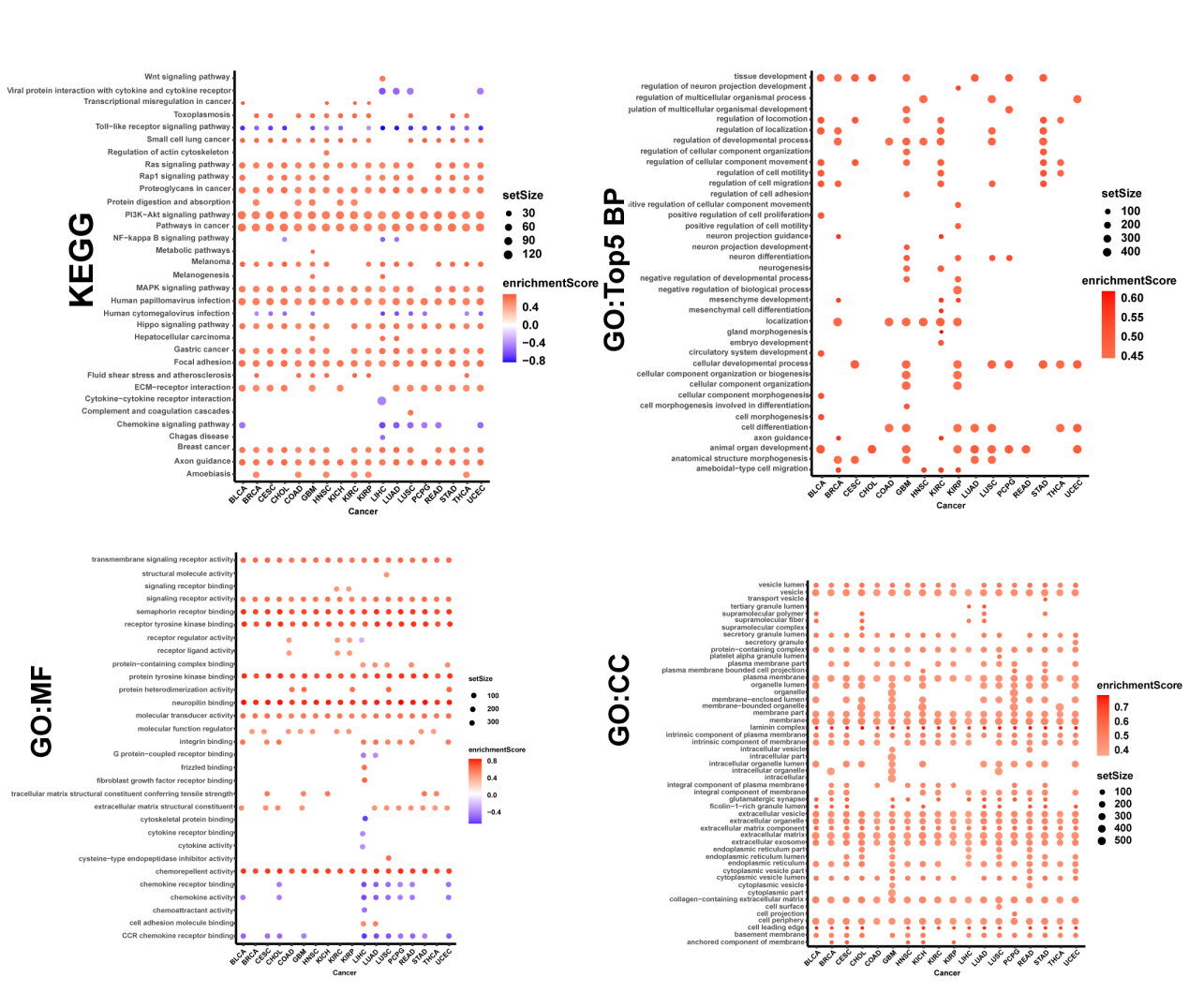


**Supplementary Figure 3.** Gene set enrichment analysis of ECM using KEGG and GO were conducted with the *ClusterProfiler* R package. BP: biological process, MF: molecular function, CC: cellular components. The results with p < 0.05 were showed; coloring based on the enrichmentscore value.


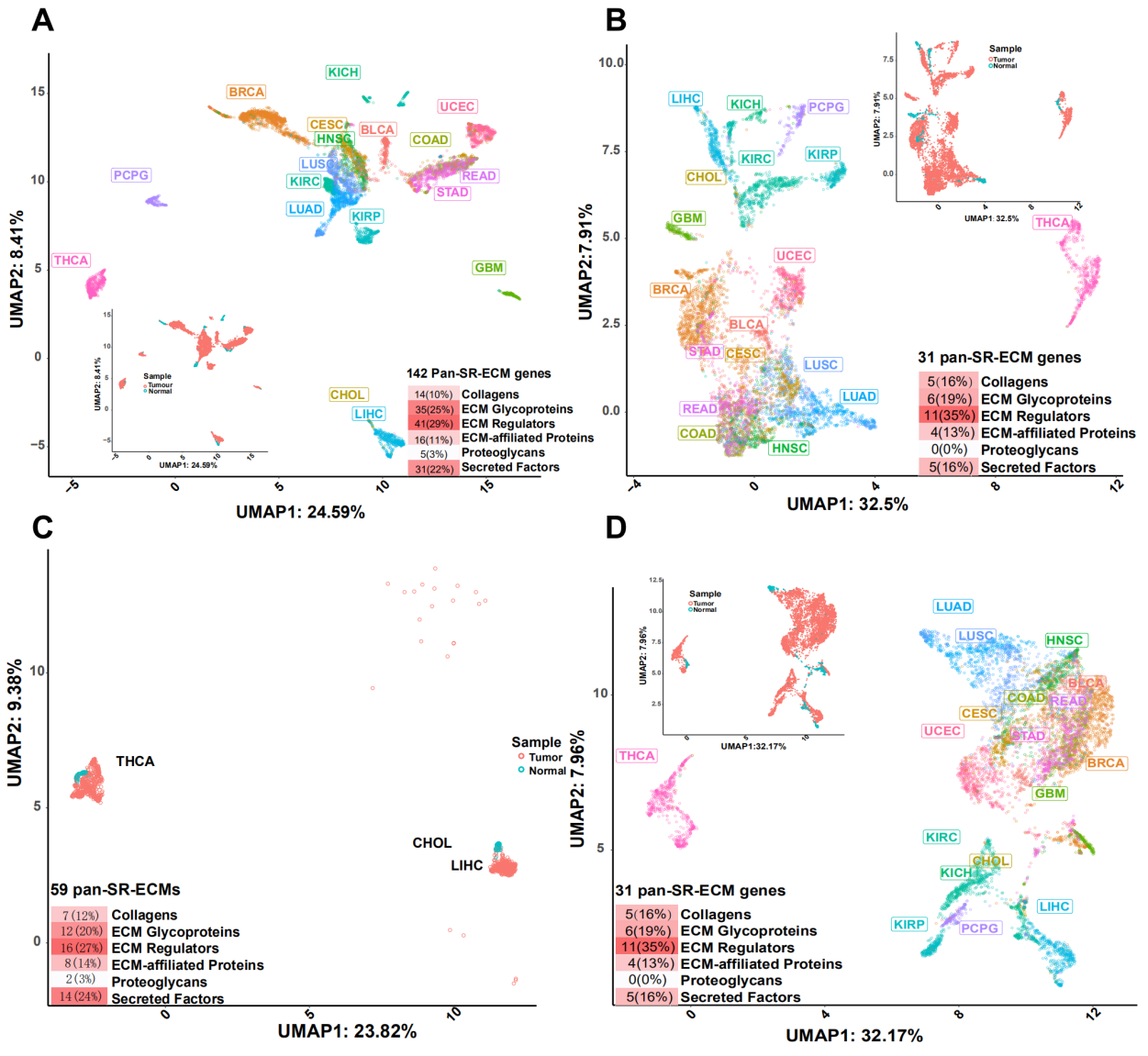


**Supplementary Figure 4**. (**A**), (**B**) UMAP analysis combined with PCA based on the log2(TPM+1) value of 170 (≥ 5 cancers types) and 31 (≥ 7 cancers types) pan-SR-ECM genes, respectively. (**C**) UMAP analysis combined with PCA based on log2(CPM+1) value of 59 pan-SR-ECM genes (≥ 6 cancers types) for THCA, CHOL and LIHC. Results show adjacent normal tissues were clustered and distinguished from the tumor tissues. (**D**) UMAP analysis combined with PCA based on log2(CPM+1) value of 31 pan-SR-ECM genes (≥ 7 cancers types). TPM: transcripts per million mapped reads. CPM: count per million mapped reads.


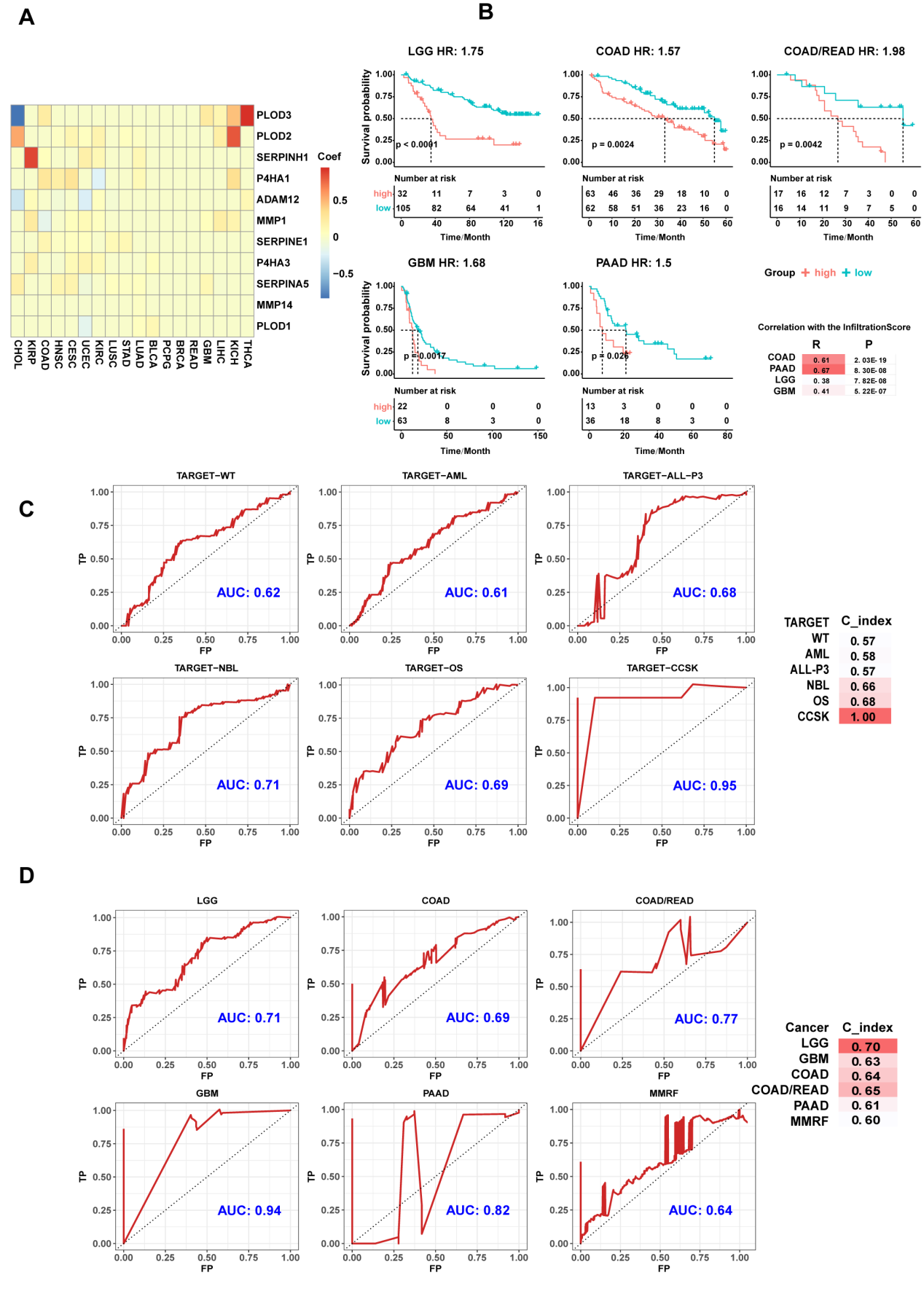


**Supplementary Figure 5**. (**A**) Coefficient for *ADAM12*, *MMP1*, *SERPINE1*, *PLOD3*, and *P4HA3* using LASSO Cox regression in 18 cancer types. (**B**) Five types of cancer were selected to verify the five gene signature in other independent data. LGG and GBM: CGGA database, COAD: GSE17536, COAD/READ: GSE71187, PAAD: GSE78229. Multi-correlation coefficient between the infiltrationscore and the signature was calculated in four types of cancer. The expression matrix of GSE71187 had been scaled, which was not appropriate to evaluate the infiltrationscore by ImmuCellAI software. R: Multi-correlation coefficient. (**C)**, **(D)** Survival results of 12 cancer types were further validated by time-dependent ROC analysis. The AUC values of all cancer types and the C_index values of most cancer types were > 0.6, which indicated favorable evidence for a combinatory gene signature. FP: false positive, TP: true positive, AUC: area under curve, ROC: receiver operating characteristic.

**
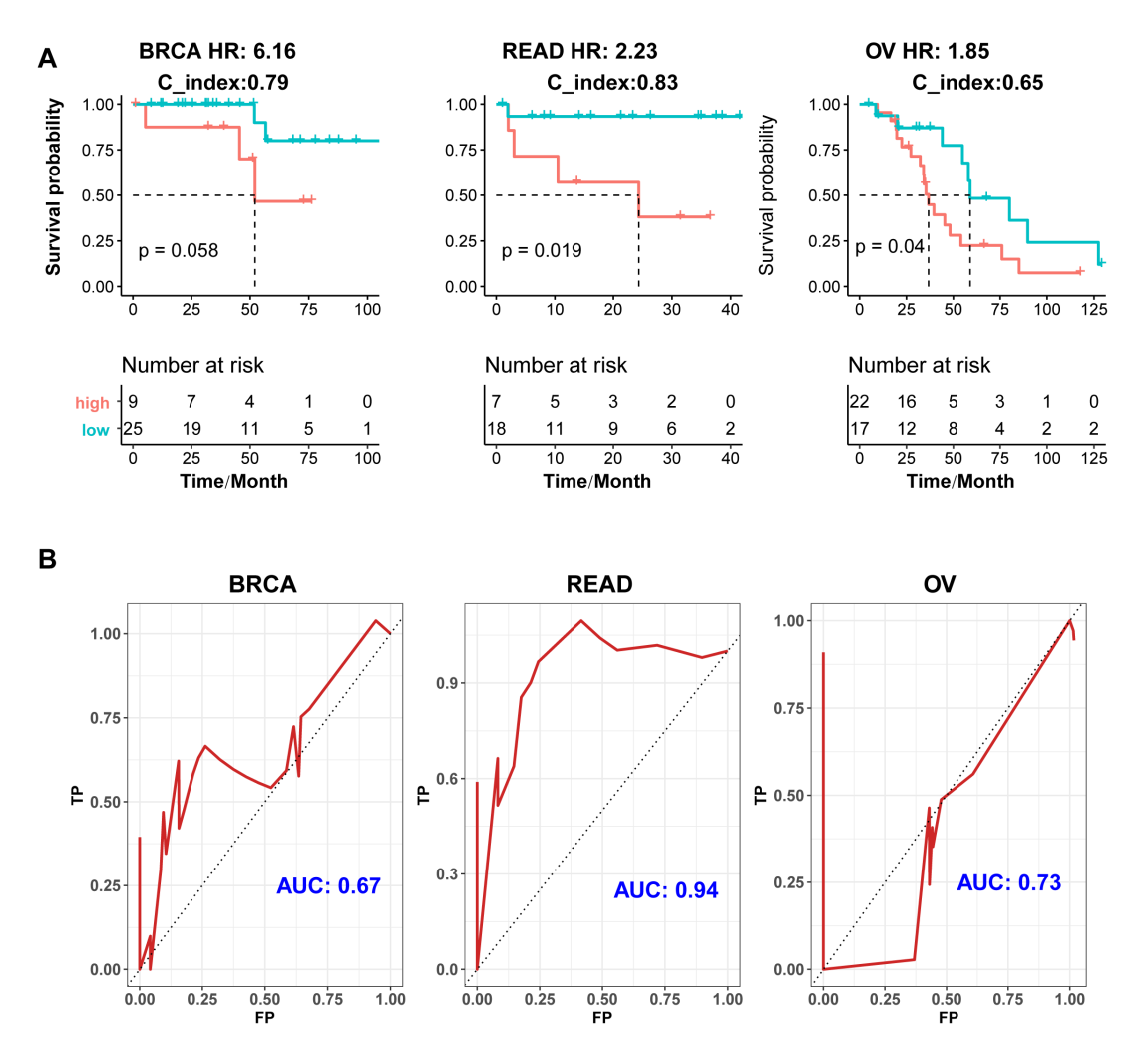
**

**Supplementary Figure 6**. **(A)**, **(B)** The prognostic performance of the signature genes was verified in proteomics using Kaplan-Meier and time-dependent ROC analysis methods, respectively. In BRCA, the five gene signature was fully detected. MMP1 and PLOD3 were detected in READ and performed survival analysis. SERPINE1 and PLOD3 were detected in OV and underwent survival analysis. BRCA, READ, and OV results were downloaded from the CPATC database; all verification databases and cancer types are different from the analytical data set.

# Supplementary Figure 7. (A)-(B), (C)-(D), (E)-(F), (G)-(H) showed IHC results of ADAM12, PLOD3, P4HA3, and SERPINE1, including the distribution and differences of IHC results compared between the tumor specimen and the adjacent normal tissues in sorts of cancer type. Higher protein expression levels of ADAM12, PLOD3, P4HA3, and SERPINE1 in multiple types of cancer were detected, which was consistent with their mRNA expression patterns. MMP1 was not available in HPA database. All data came from the Human Protein Altas database. IHC: immunohistochemistry, T: tumor tissue, N: normal tissue.


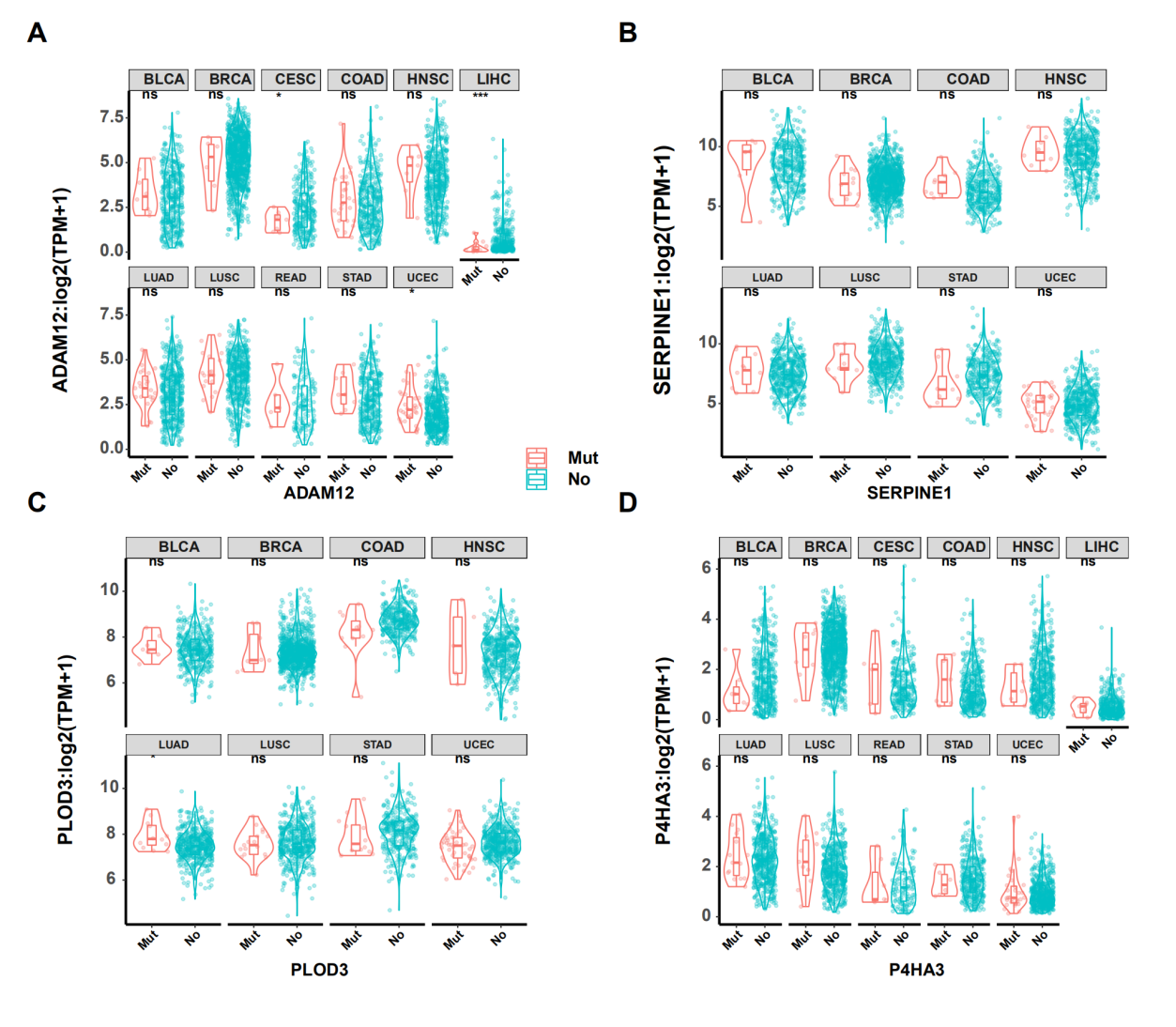


**Supplementary Figure 8**. (**A**)**-**(**D**) The influence of mutations on *ADAM12*, *SERPINE1*, *PLOD3*, and *P4HA3* were compared in mutational- and wild-type tumor tissues across various cancer types. The p value was calculated using the Student’s t-test. Mutational information was not calculated for *MMP1.* TPM: transcripts per million reads.


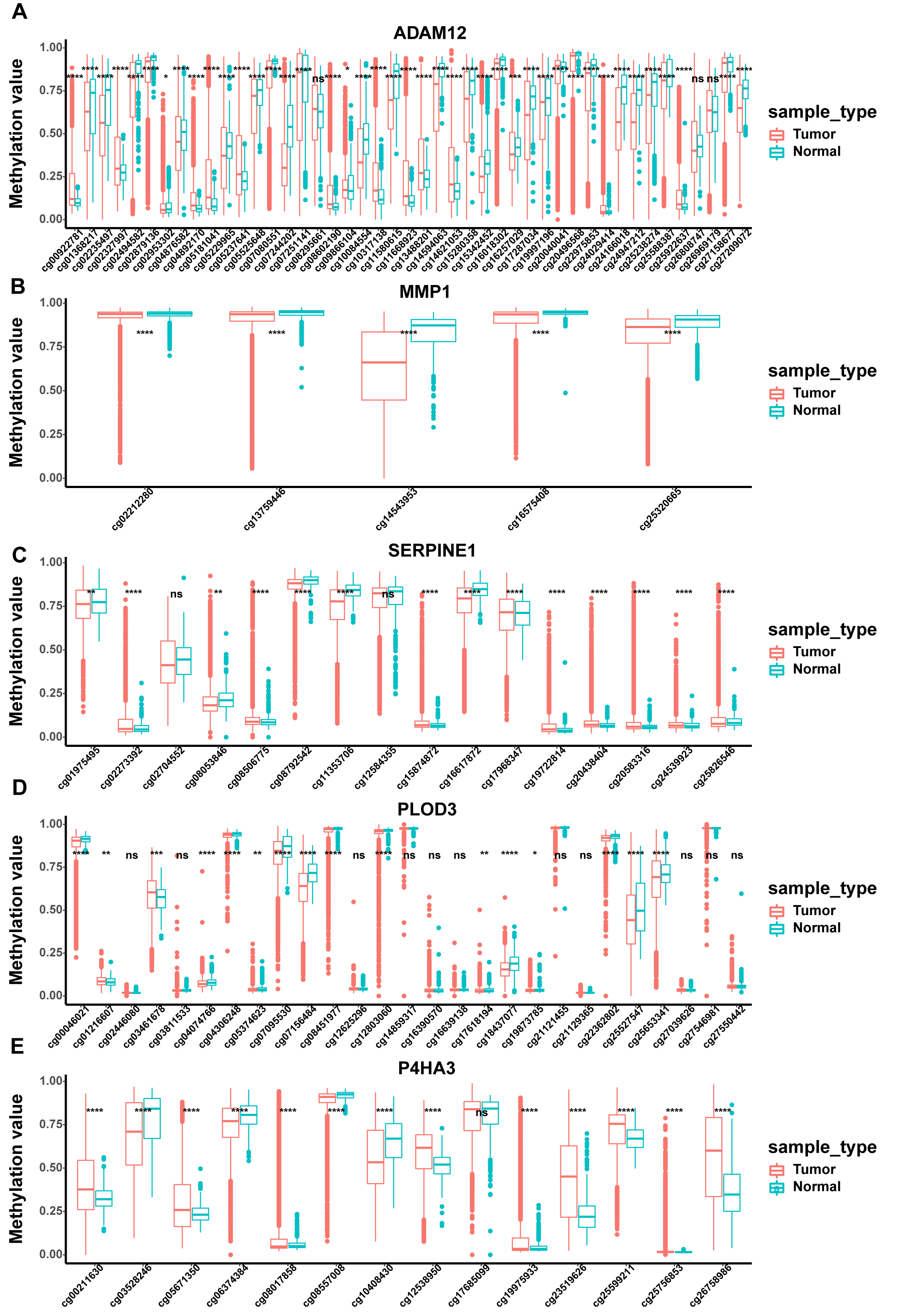


**Supplementary Figure 9.** (**A**)**-**(**E**) Methylation levels of *ADAM12*, *MMP1*, *SERPINE1*, *PLOD3*, and *P4HA3* compared between tumor and adjacent normal tissues. The p value was calculated using Student’s t-test.


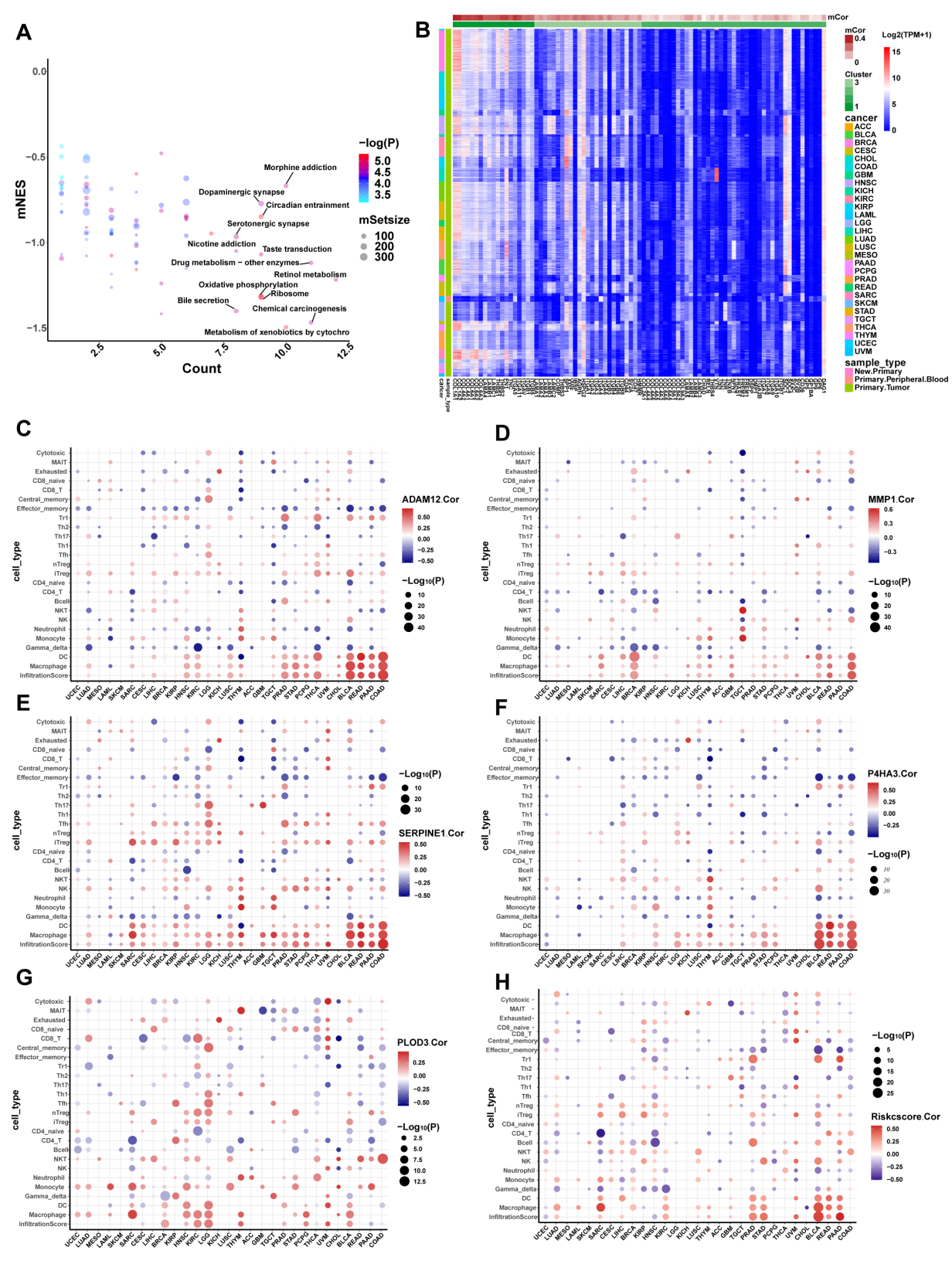


**Supplementary Figure 10.** (**A**) Distribution of the significantly enriched KEGG pathways with mNES < 0; x axis: the count of the KEGG pathway with p.adjust <0.05, y axis: mNES, the mean normalized enrichmentscore of a KEGG pathway with p.adjust <0.05. Coloring is based on the minimum p.adjust. (**B**) Expression levels of the ECM-receptor interaction gene set profile in 26 cancer types. (**C**)**-**(**H**) Correlation coefficients of *ADAM12*, *MMP1*, *SERPINE1*, *PLOD3*, *P4HA3* and risks core with infiltration score and the abundance of types of immune cells. The list was ordered based on the correlation coefficient with infiltration score. Results with p < 0.05 are shown. TPM: transcripts per million mapped reads.
